# A comparative study of single and double incision for L4/5 and L5/S1 double-level percutaneous interlaminar lumbar discectomy

**DOI:** 10.3389/fsurg.2022.955987

**Published:** 2022-08-30

**Authors:** Yingchuang Tang, Zixiang Liu, Hao Liu, Junxin Zhang, Xiaoyu Zhu, Zhonglai Qian, Huilin Yang, Haiqing Mao, Kai Zhang, Hao Chen, Kangwu Chen

**Affiliations:** ^1^ Department of Orthopedic Surgery, The First Affiliated Hospital of Soochow University, Suzhou, China; ^2^ Department of Orthopedic Surgery, Affiliated Hospital of Yangzhou University, Yangzhou, China

**Keywords:** single/double incision, interlaminar approach, adjacent double lumbar disc herniation, endoscopic spinal decompression, minimally invasive spine surgery

## Abstract

**Objective:**

This study aims to investigate the clinical outcome of single and double incision for double-level percutaneous interlaminar lumbar discectomy

**Methods:**

A retrospective analysis was performed involving patients with L4/5 and L5/S1 double-level lumbar disc herniation who received percutaneous interlaminar lumbar discectomy (PEID) in our hospital from January 2017 to December 2020. These enrolled patients were divided into single- and double-incision groups, with 25 patients in each group. We compared the incision length, operation time, fluoroscopy times, and length of hospital stay between the two groups. Meanwhile, the postoperative visual analogue scale (VAS), Oswestry Disability Index (ODI), Japanese Orthopedic Association score (JOA), and modified MacNab standard were used to evaluate the outcomes of the patients within the two groups.

**Results:**

It showed that the single-incision group performed better than double-incision group in incision length, operation time, and fluoroscopy times (*P *< 0.001). The VAS score, JOA score, and ODI index in the two groups were significantly decreased at the time points of postsurgery, 1 month after surgery, and the last follow-up (*P *< 0.01), but there was no statistical significance between the two groups involving above parameters (*P *> 0.05). At the last follow-up, the excellent and good rates of MacNab efficacy in the two groups were 92% and 88%, respectively, but no significant difference was observed between the two groups (*P *> 0.05).

**Conclusion:**

Both the single- and double-incision approaches are effective and safe for managing L4/5 and L5/S1 double-level LDH. Single-incision PEID for treating L4/5 and L5/S1 double-segment lumbar disc herniation has advantages of less trauma, fewer intraoperative fluoroscopy times, and shorter operation time, as compared to double-incision PEID. However, the operation of double-segment LDH through a single laminar incision is difficult, the learning curve is steep, and professional skill is highly required. Importantly, the surgical indications should be strictly grasped.

## Introduction

Lumbar intervertebral disc herniation (LDH), a common degenerative disease of the lumbar spine, usually occurs at a single level (1). It was mainly presented with the clinical symptom of lumbar and leg pain, seriously affecting patients' daily life (1, 2). In the clinic, we find that it is not rare for young patients to develop double-level LDH, while patients who fail to receive stepwise conservative treatment always need further surgical interventions (3, 4). Lumbar discectomy is the traditional treatment for LDH, but it has some disadvantages, such as difficulty in operation skills and resection of normal structures, including skeletal tissue (5, 6). With the continuous development of minimally invasive spinal techniques, percutaneous endoscopic interlaminar discectomy (PEID) and microendoscopic discectomy (MED) have been widely used in clinical practice (7, 8). Compared with traditional surgery, PEID and MED are characterized by less trauma, less bleeding, faster recovery, and less impact on lumbar stability. Despite the rapid development of PEID, its efficacy in the management of symptomatic double-level LDH remains controversial (9–11). The rate of double-level lumbar disc herniation is relatively low, and the open lumbar discectomy or double-incision PELD is mostly used. Recently, we found that the single-incision translaminar approach for L4/5 and L5/S1 double-segment LDH can also achieve a satisfactory effect, but there is no consensus on which approach is better. Therefore, we aim to compare the clinical outcomes of single- and double-incision PEID for treating L4/5 and L5/S1 double-level LDH.

## Materials and methods

### Patients

The following inclusion criteria are applied: (1) ipsilateral lumbar disc herniation in L4/5 and L5/S1, two adjacent levels, as confirmed by computed tomography (CT) and magnetic resonance imaging (MRI); (2) definite history of lumbar and leg pain with neurological symptoms and signs; (3) symptoms and signs consistent with the images; (4) failure of conservative treatment for more than 3 months; and (5) at least 12 months of follow-up data available. The exclusion criteria are as follows: (1) patients with cauda equina syndrome or progressive neurological impairment requiring emergency surgery; (2) with spinal instability and spinal canal stenosis; (3) nonadjacent level of LDH; (4) patients with cephalic overdissociation of L4/5 nucleus pulposus and caudal overdissociation of L5/S1 nucleus pulposus; and (5) previous surgery involving the lumbar spine, concomitant somatic, or psychological conditions, such as uncontrolled myocardial ischemia, diabetes, spinal tumor, fracture, or infection.

According to the criteria, 50 patients with adjacent double-segment LDH (L4/5 and L5/S1) who received two-level PEID surgery in our hospital from January 2017 to December 2020 were enrolled. The patients were divided into a single-incision group (25 patients) and a double-incision group (25 patients). The patients were informed of the advantages and disadvantages of the two surgical options. Meanwhile, they were instructed that there was no sufficient evidence-based medicine showing which surgical option was better.

### Surgical technique

Both groups were performed by the same surgical team. In the single-incision group, after successful general anesthesia, the patient was prone on the operating table, C-arm fluoroscopy was positioned in the middle of the L5 vertebral body, and L5/S1 and L4/L5 intervertebral spaces were marked. An incision of about 6 mm was made at the midpoint of the gap. The angle was adjusted to puncture into the L4/L5 intervertebral space, and a blunt dilator was inserted before placing a working sheath. After the dilator was removed, an endoscope was placed in the external working sheath. The ligamentum flavum was cut diagonally and layer by layer in 3–5 mm to expose the spinal canal contents. Part of the transparent adipose tissue was removed to reveal the dural sac, and the endoscope channel and external working sheath were adjusted to explore the nerve root position. During the operation, a radiofrequency ablation electrode was used to stop bleeding. The disc was exposed after the nerve root was pushed and protected. Nucleus pulposus was obtained alternately with different nucleus pulposus forceps. The L5/S1 intervertebral space was entered from the same puncture point adjustment angle, and the L5/S1 intervertebral disc was treated with endoscopic nucleus pulposus resection in the same way.

In the double-incision group, after successful general anesthesia, the patient was prone on the operating table, C-arm fluoroscopy was positioned at L5/S1 and L4/L5, respectively, and two incisions of about 6 mm in length were made in the middle of L5/S1 and L4/L5. First, L4/L5 disc nucleus pulposus was removed with the same incision. After the completion of L4/L5 discectomy, the L5/S1 intervertebral space was punctured, and endoscopic nucleus pulposus resection was performed on the L5/S1 intervertebral disc in the same way.

### Clinical evaluation

Both groups were followed up for at least 12 months, with an average of 15.20 ± 2.06 months and 15.92 ± 2.64 months, respectively. The incision length, operation time, fluoroscopy time, and hospital stay were recorded. The visual analogue scale (VAS) was used to evaluate the low back and leg pain, and the Oswestry disability index (ODI) was used for the evaluation of the functional disability. Both VAS and ODI were collected at preoperation, 1-month postoperation, 3-month postoperation, and the last follow-up time. The modified MacNab efficacy standard was applied to evaluate the final outcome of patients, which were divided into excellent, good, fair, and poor.

### Statistical analysis

Statistical analysis was performed using SPSS (Statistical Package for Social Sciences) version 21.0 (IBM SPSS, Chicago, IL, USA) software. Continuous variables are presented as mean ± SD and compared by Student's *t*-test. Categorical data are expressed as the number (percentage) and compared by Pearson's chi-squared test. Significance was set at *P* < 0.01 or *P* < 0.05.

## Results

All 50 patients were successfully operated on and had at least 12 months of follow-up (range 12–22 months). The key demographic baseline parameters and follow-up time are summarized in Table 1.

**Table 1:** Baseline patient characteristics between the two groups.

Variable	Single-incision group	Double-incision group	*P* value
*N*	25	25	–
Gender (M/F)	18/7	20/5	0.508
Age (year)	32.60 ± 4.74	31.32 ± 5.46	0.380
Course of disease (months)	5.88 ± 1.64	6.32 ± 1.84	0.377
Follow-up (months)	15.20 ± 2.06	15.92 ± 2.64	0.288

Values are expressed in mean ± standard deviation.

The operative surveys between the two groups are shown in Table 2. In general, significant improvements were observed in leg and back pain after surgery in both groups. Compared to that in the double-incision group, the average incision length in the single-incision group was much shorter (5.91 ± 0.68 mm vs. 11.72 ± 1.36 mm, *P* < 0.001). Moreover, the average operation time was faster (81.84 ± 15.79 vs. 94.28 ± 12.59 min, *P* < 0.01) and the average fluoroscopy time was significantly decreased (3.64 ± 1.90 vs. 7.72 ± 1.40, *P* < 0.001) in the single-incision group. Furthermore, the average length of hospital stay in the single-incision group was less than that in the double-incision group (3.48 ± 0.81 vs. 3.44 ± 0.58 days, *P* = 0.810). The results indicated that the single-incision group has a shorter incision length, faster operation time, and fewer intraoperative fluoroscopy time.

**Table 2:** Comparison of intraoperative outcomes between the two groups.

Variable	Single-incision group	Double-incision group	*P* value
Incision length (mm)	5.91 ± 0.68	11.72 ± 1.36	<0.001
Operative time (min)	81.84 ± 15.79	94.28 ± 12.59	<0.01
Frequency of fluoroscopy	3.64 ± 1.90	7.72 ± 1.40	<0.001
Hospital stays (days)	3.48 ± 0.81	3.44 ± 0.58	0.810

Values are expressed in mean ± standard deviation.

The functional improvement of the patients was satisfactory. The postoperative changes in the JOA score within the two groups are shown in Table 3. For the single-incision group, the mean JOA scores improved from 12.20 ± 2.12 at preoperative to 23.72 ± 3.78 at 1 month postoperatively (recovery rate 69.50 ± 18.95%), further increased to 27.08 ± 1.55 at 3 months postoperatively (recovery rate 88.63 ± 9.09%). In the double-incision group, the mean JOA scores improved from 13.26 ± 2.30 at preoperative to 23.20 ± 4.00 at 1 month postoperatively (recovery rate 65.08 ± 21.86%), further increased to 27.24 ± 1.17 at 3 months postoperatively (recovery rate 89.17 ± 7.07%). At the last follow-up, the mean recovery rates of the two groups were 93.39 ± 6.44% and 93.15 ± 5.92%, respectively. There was no statistical difference in the JOA score and associated recovery rate between the two groups (*P* > 0.05).

**Table 3:** Comparison of JOA score results between the two groups.

Variable	Single-incision group	Double-incision group	*P* value
Mean JOA score			
Preop	12.20 ± 2.12	13.26 ± 2.30	0.800
1 month postop	23.72 ± 3.78*	23.20 ± 4.00*	0.639
3 months postop	27.08 ± 1.55*,**	27.24 ± 1.17*,**	0.682
Last follow-up	27.88 ± 1.09*,**,***	27.84 ± 1.11*,**	0.898
Mean recovery rate^a^			
1 month postop	69.50 ± 18.95	65.08 ± 21.86	0.449
3 months postop	88.63 ± 9.09**	89.17 ± 7.07**	0.815
Last follow-up	93.39 ± 6.44**,***	93.15 ± 5.92**,***	0.891

Values are expressed in mean ± standard deviation.

JOA, Japanese Orthopaedic Association; Preop, preoperative; Postop, postoperative.

^a^ Mean recovery rate (%) = (postoperative JOA score − preoperative JOA score)/(29 − preoperative JOA score) × 100%.

* *P* < 0.01 compared to the preoperative value.

** *P* < 0.05 compared to the 1-month postoperative value.

*** *P* < 0.05 compared to the 3-month postoperative value.

The postoperative scores of VAS and ODI in both groups were significantly decreased compared with those before the operation (Table 4). Symptoms continued to improve at different time points after surgery in both groups. There was no significant difference between the two groups involving VAS and ODI scores at different time points after surgery. At the last follow-up time, the overall excellent/good rate was 92% in the single-incision group (23/25) and 88% in the double-incision group (22/25), and there was no significant difference between the two groups (Table 5).

**Table 4:** Comparison of VAS and ODI score results between the two groups.

Variable	Single-incision group	Double-incision group	*P* value
Mean VAS score			
Preop	7.04 ± 1.13	7.20 ± 1.00	0.599
1 month postop	1.92 ± 1.08*	2.12 ± 0.93*	0.485
3 months postop	1.20 ± 0.91*,**	0.84 ± 0.80*,**	0.145
Last follow-up	0.40 ± 0.65*,**,***	0.28 ± 0.54*,**,***	0.480
Mean ODI score			
Preop	70.24 ± 4.05	70.08 ± 3.70	0.612
1 month postop	24.08 ± 4.45*	22.16 ± 4.47*	0.135
3 months postop	17.04 ± 3.96*,**	15.92 ± 4.02*,**	0.326
Last follow-up	9.92 ± 2.86*,**,***	9.84 ± 3.36*,**,***	0.928

Values are expressed in mean ± standard deviation.

VAS, visual analogue scale; ODI, oswestry disability index scores.

* *P* < 0.01 compared to the preoperative value.

** *P* < 0.05 compared to the 1-month postoperative value.

*** *P* < 0.05 compared to the 3-month postoperative value.

**Table 5:** Modified MacNab criteria results.

Variable	Single-incision group	Double-incision group	*P* value
Modified MacNab			
Excellent	20	19	
Good	3	3	
Fair	2	2	
Poor	0	1	
Excellence/good rate (%)	92	88	0.795

All wounds healed after the first intention. In the double-incision group, one patient developed L5/S1 segment recurrence 6 months after surgery and underwent an open lumbar discectomy according to the patient's requirements. No recurrence occurred in the single-incision group. There was no significant difference in terms of recurrence between the two groups (*P* > 0.05). All of the patients ultimately acquired back and leg pain relief.

**Figure 1:**
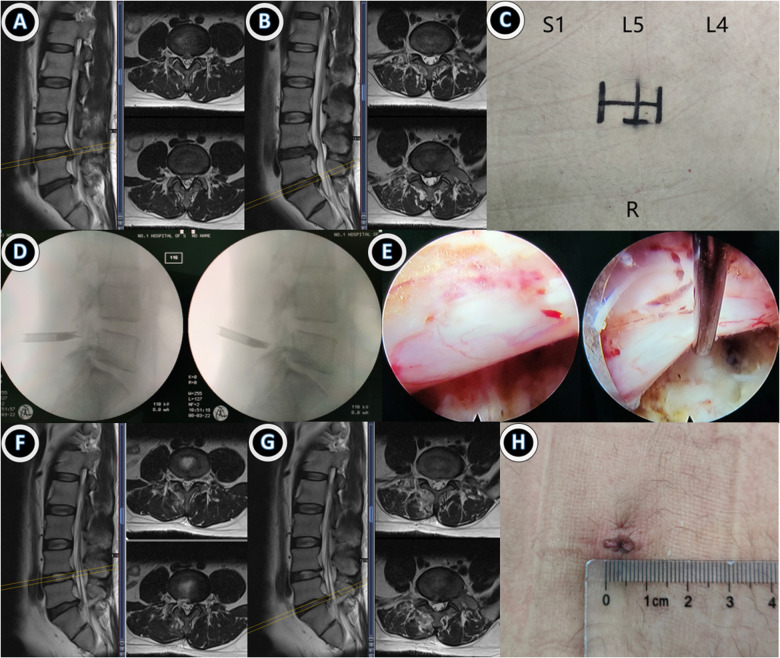
Case 1: A 38-year-old male patient with ipsilateral disc herniation at L4/5 and L5/S1. Endoscopic double-segment discectomy was performed through a single-incision and interlaminar approach. (**A,B**) Preoperative lumbar MRI suggested disc herniation at L4/5 and L5/S1 levels; (**C**) preoperative single-incision design; (**D,E**) intraoperative double interstitial working tubes were successively placed for nuclear pulposus excision, and the nerve root relaxation was observed under a microscope; (**F,G**) MRI review at 1 month after surgery suggested that the protrusion was completely removed; (**H**) Postoperative incision was about 7 mm, and MRI re-examination 3 months after surgery showed no further protrusion.

**Figure 2:**
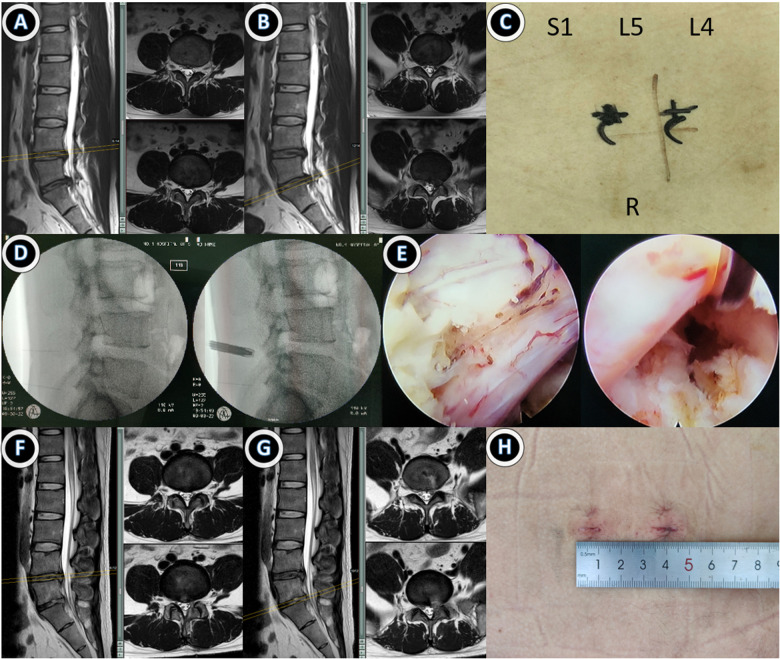
Case 2: A 28-year-old male patient with ipsilateral disc herniation at L4/5 and L5/S1. Endoscopic double-segment discectomy was performed with double incision through an interlaminar approach. (**A,B**) Preoperative lumbar MRI suggested disc herniation at L4/5 and L5/S1 levels; (**C**) preoperative double-incision design; (**D,E**) intraoperative double interstitial working tubes were successively placed for nuclear pulposus excision, and the nerve root relaxation was observed under a microscope; (**F,G**) MRI review at 1 month after surgery suggested that the protrusion was completely removed; (**H**) Postoperative incision was about 16 mm, and MRI re-examination 3 months after surgery showed no further protrusion.

Typical cases of the single- and double-incision groups are shown in Figures 1, 2. There was no dural laceration, nerve root injury, cerebrospinal fluid leakage, infection, postoperative paresthesia, or other serious complications in either of the groups.

## Discussion

Minimally invasive techniques have been widely used in treating lumbar disc herniation in the past decade. Single-incision treatment of two-segment LDH is a minimally invasive operation with a small incision, less trauma, quick effect, early ground operation, and other characteristics. In this study, both groups of patients achieved good clinical results after surgery, and the quality of life was obviously improved. In addition, the postoperative JOA score, VAS score, and ODI index were significantly lower than those before surgery. The excellent and good rates of the modified MacNab in the two groups at the last follow-up were 92% and 88%, respectively, and there was no significant difference between the two groups (*P* > 0.05), which indicated the effectiveness of these two surgical methods. In our study, no nerve root injury, cerebrospinal fluid leakage, infection, and postoperative lower limb paresthesia occurred in the two groups, and the results showed that these two minimally invasive surgical methods have high safety and few complications. Single-incision endoscopic spinal treatment of two-segment LDH through an interlaminar approach can reduce the incision length, radiation frequency, and operation time compared with double-incision treatment, and the single-incision treatment is more aesthetic than double-incision treatment.

The health effects of fluoroscopic radiation are also of concern to surgeons and patients (12). Compared with single incision, double incision requires multiple catheterizations. However, once the puncture quantity is increased, it is inevitable to increase the number of fluoroscopies, which also increases the operation time and radiation exposure to doctors and patients. Radiation exposure has been linked to an increased risk of cancer, cataract, and cardiovascular disease (13, 14). Therefore, a single incision can effectively reduce the number of fluoroscopy and surgical time, reducing the radiation exposure of doctors and patients.

The most common cause of failure in minimally invasive or endoscopic spine surgery is incomplete excision or intraoperative complications (15–17). In the double-incision group, a patient with recurrent pain in the lower extremity was discharged after a second operation. There were no serious complications such as dural injury in both groups. Surgical puncture is the difficulty of operation but also the key point for the successful completion of the operation in handling double segments by single incision. The laminar space of L5/S1 is larger than that of L4/5, and the puncture point could be slightly closer to L4/5 due to the need to remove the part bone of the facet and lower edge of the lamina (18). The surgeons should accurately determine the location of nerve roots during operation to avoid nerve root injury. If the disc is adherent to the nerve root or the dural sac, the disc should not be released forcibly. If necessary, open surgery should be performed (19). The nucleus pulposus tissue that can be removed should be removed as far as possible; otherwise, with the postoperative activities of the patient, the residual nucleus pulposus is easy to shift again and cause compression, resulting in disease recurrence. One incision should be used as far as possible, but it should be based on the intraoperative situation. A single incision should not be forced; otherwise, it may lead to incomplete decompression (15). Double incision usually facilitate better working pipe placement and decompression. Single-incision treatment for two-segment LDH increases the probability of nerve root injury during puncture, so a single incision cannot be forced in two-segment surgery. In addition, a single incision is not suitable for bilateral lumbar disc herniation. Not only can surgeons understand the type of disc herniation through preoperative imaging tests such as x-rays, CT scans, and MRI but also they can understand the feasibility of endoscopic surgery (20). The degree of disc herniation, degree of migration, severity of adhesion, risk of dural tear, the softness of the herniated disc, and concurrent spinal stenosis should be assessed. In addition, the use of a single incision to deal with double intervertebral disc increases the difficulty of operation, so the operator needs to strictly grasp the indications and try to use a single incision for the treatment of two-level disc herniation on the basis of mastering the two-level incision (21, 22).

In conclusion, the endoscopic percutaneous interlaminar approach by single incision for the treatment of two-level lumbar disc herniation is feasible and safe. Compared with double incision, single incision exerts less trauma, shorter incision, and faster postoperative recovery. However, due to the difficulty of operation, it is necessary to strictly grasp the surgical indications and possess certain experience in single-segment endoscopic surgery. Postoperative functional exercise guarantees a curative effect. In the future, the indications of endoscopic percutaneous interlaminar approach will be further expanded, and precision will be the inevitable trend.

The study has some limitations. First, the study was not a double-blind randomized controlled trial. Surgeons and patients have different perceptions of treatment and prognosis, which may influence outcome assessment. Second, the surgeon's preference for surgical technique may also influence the outcome. Finally, this study was a single-center study with a short follow-up period. The comparison of postoperative clinical efficacy of LDH requires high-quality multicenter and long-term follow-up studies.

## Conclusion

In conclusion, the single-incision approach has more advantages in operation time, incision length, and fluoroscopic time but exerts no difference in terms of JOA, VAS, and ODI scores or postoperative complications as compared to the double-incision approach.

## Data Availability

The original contributions presented in the study are included in the article/Supplementary Materials, further inquiries can be directed to the corresponding author/s.
